# Factors influencing the care provided for periviable babies in Australia: a narrative review

**DOI:** 10.1186/s12978-015-0094-8

**Published:** 2015-11-25

**Authors:** Susan Ireland, Robin Ray, Sarah Larkins, Lynn Woodward

**Affiliations:** 1 The neonatal unit, The Townsville Hospital, 100 Angus Smith Dve, Douglas, Queensland 4814 Australia; 2 College of Medicine and Dentistry, James Cook University, Douglas, Queensland 4814 Australia

**Keywords:** Prematurity, Periviable, Resuscitation, Decision-making, Ethics, Parental autonomy, Australia

## Abstract

Survival at extreme prematurity is becoming increasingly common. Neurodisability is an increasing risk with decreasing gestation. This review outlines the risks of extreme prematurity and the attitudes of health care providers and families in Australia of periviable babies. High quality data is difficult to find due to differing definitions and methods of assessment of disability. Meta-analyses of outcomes of prematurity published from 2008 to 2013, including babies born from 1990 onwards, suggest a severe disability rate of around 20 % at 22 to 26 weeks completed gestation, with moderate disability decreasing with increasing gestation. Studies show that Australian health care providers underestimate the survival and positive outcomes of these babies. The majority of Australian health care providers state that parental preference would determine the decision to offer care to babies at 23 weeks gestation, however, all had a threshold above which parental preference would be ignored in favour of resuscitation .This ranged from 22 to 27 completed weeks gestation. The few studies examining Australian parental involvement in resuscitation decisions, showed that the majority of parents felt that health professionals alone had made the decision to resuscitate their extremely preterm babies and the parents themselves did not wish to be the primary decision makers in withholding care. The babies progressed better than parents had expected following antenatal counselling. The attitudes of health care providers, experiences and opinions of parents seem to be at odds with the current move to increase parental decision making at the most extremes of gestation. Current Australian guidelines suggest parental decision making below 25 weeks gestation, and primarily clinician decision making over this gestation. The increased risks of prematurity and adverse outcomes for the North Queensland population is also explored. This population has a high proportion of Aboriginal and Torres Strait Islanders who have increased risks which are primarily linked to poor socioeconomic factors and are highest for the most remote residents. Attitudes towards delivery of care to these highest risk babies from health professionals and in the populations themselves have not been studied.

## Background

Australia is a wealthy country where a high level of neonatal intensive care is available for all its residents without direct financial charge. Care is provided for babies under 32 weeks gestation in centralized tertiary intensive care units. Technological changes in the field of neonatology have lead to the survival of increasingly premature neonates [1–3] leading to the current age of periviability, which is generally considered to be 22 to 26 completed weeks of gestation [4].

Premature delivery before 37 completed weeks of gestation occurs in 8.3 % of Australian pregnancies [5]. Delivery from 20 to 27 weeks gestation is known to occur in 0.8 % of deliveries in Australia [5], which includes stillbirths and pre-viable babies. Within these statistics, the exact figures for periviability between 23 and 26 completed weeks gestation are difficult to determine due to the method of capturing data. The use of antenatal steroids in women with pregnancies at risk of early delivery, and the development of artificial surfactant, have been major advances which have lead to an improvement in respiratory wellbeing [6] and survival. Survival rates of 50–80 % for babies at 23 to 26 weeks gestation are expected in tertiary neonatal units [3, 7, 8]. However, survival may come at a cost of a significant risk of long term neurological morbidity, exhibited as intellectual impairment, cerebral palsy and sensory impairment [9–11]. Studies of long-term outcomes are scarce in the Australian context, but meta-analyses of large international studies suggest a risk of severe disability of approximately 20 % below 27 weeks gestation [12, 13].

Recent discoveries have lead to management which reduces the complications that occur after birth. These include the use of magnesium sulphate which is given to mothers prior to delivery and which has been shown to reduce cerebral palsy [14]. Probiotics, when given to the extremely preterm newborn, have been shown to reduce necrotizing enterocolitis, which is a major risk factor for long term neurological morbidity [15]. However, there has not been sufficient time to evaluate the long term effects of these changes on morbidity.

Whilst the long-term goal of neonatal care is to produce healthy infants, the early clinical intensive care course of the extremely preterm neonate is difficult and a degree of suffering is inevitable. Parents of less premature babies describe the stress of the neonatal intensive care and perceive that there is pain and suffering [16, 17]. At discharge from hospital, the parents will then become responsible for the future care of babies, who may be left with sequelae following the provision of this care. The early suffering of the periviable baby, as well as the potentially severe life long morbidity are factors which need to be considered when deciding to offer these babies life sustaining intensive care.

This review aims to outline the outcomes of extreme prematurity and the perspectives of health care providers and families of periviable infants in Australia.

## Review

### Methods

**Fig. 1:**
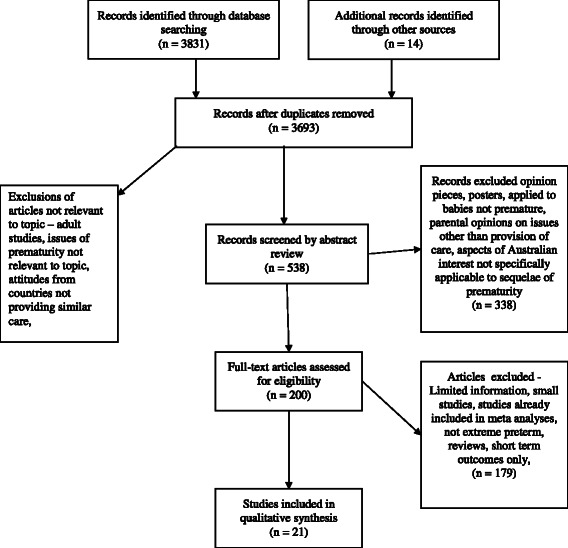
Flow diagram of literature search

A search was performed using PubMed, Medline, CINAHL and Google Scholar to identify articles exploring the outcome of perinatal care, resuscitation guidelines, parental perspectives, health care perspectives and Australian specific literature around extreme prematurity. Key words used (including combinations and relevant truncated words and phrases) included ‘premature’, ‘preterm’, ‘periviable’, ‘neonatal resuscitation guidelines’, ‘Australia’, ‘rural’, ‘disabled’, ‘child’, ‘ethics’, ‘parents’. In addition, the search was expanded using references found in the articles identified and other articles citing them. Local and government publications were searched for relevant statistical information. The search was limited to English language publications from 1985 to 2014. As antenatal steroids, artificial surfactant and other technology was unavailable prior to 1985, studies done before this era do not reflect the current attitudes towards periviability because outcomes were less positive. Initially 3693 references were obtained. Most of these were rejected prior to further screening as they clearly had no relevance to the literature review itself, for example adult studies and studies looking at other aspects of prematurity. After applying the inclusion and exclusion criteria agreed on by the authors, the abstracts of the remaining 538 articles were reviewed by the first author. Of these, 338 papers were excluded on the basis that they included opinion pieces, posters, applied to babies who were not premature or were parental perspectives on issues other than periviability. The remaining 200 full text articles were accessed. Articles were then excluded where they were reviews or provided limited information in single small center studies except where innovative design was used. Guidelines were included where they pertained to Australia or similarly structured neonatal models of care. 21 articles are discussed in this review. This includes the two meta analyses of outcomes, all seven articles reflecting medical and parental opinions in Australia, seven with data pertaining to rural children with disability and five specifically to the population in North Queensland. See Fig. 1. See Table 1.

**Table 1:** Table of papers found outlining opinions of medical personnel and families in Australia with regards to the provision of care for extremely preterm babies

Study author	Date	Population	Sample size	Methodology	Outcome of study
Mulvey et al. [38]	Published 2001	Obstetricians in hospitals with Level 3 NICU, No Northern Australian participants	89 participants, 48 % response rate	Survey	Majority would always discuss resuscitation from 23 weeks. Majority underestimate survival. Paediatric opinion then parental opinion used to inform decisions.
Gooi et al. [39]	Published 2001	Obstetricians from hospitals providing level 2 neonatal care	174 participants, 75 % response rate	Survey	Median for resuscitation 24 weeks gestation. Refer to tertiary unit over 24 weeks except in West Australia and Victoria – 23 weeks
De Garis et al. [36]	Published 1987	Neonatologists from all 18 NICU in Australia	51 participants, response rate not given	Survey, some open ended questions	Majority under estimate survival. Majority offer full resuscitation over 24 weeks gestation, consider later withdrawal of care if neurological concern
Oei et al. [40]	Study 1997–1998 Published 2000	All neonatologists in Australia	71 participant neonatologists 93 % response rate , 41 neonatal nurse participants, 74 % response rate	Survey, some open ended questions	Doctors median age for care 24 weeks- range 22–25 Nurses median age of care 25 weeks- range 23–28 Parental opinion should influence resuscitation but majority would overrule parents at 25 weeks Doctors more accurate estimate of survival and morbidity
Munro et al. [37]	Published 2001	100 neonatologists in Australia	70 % response rate	Survey	Majority always counseled over 23 weeks and would give mortality and morbidity data. Obstetricians’ main influence in decision to provide resuscitation. Consider parental opinion from 23 to 25 weeks
Martinez et al. [43]	Study 1999 Published 2005	Part of large Pacific Rim study comparing practice in different countries. Neonatologists throughout Australia	Participant number unclear, 68 % response rate	Survey	Obstetric opinion and previous parental infant loss would be main influences of what counseling provided. Majority said that family should be decision makers for resuscitation where parents and doctor disagreed
Partridge et al. [26]	Study 1998 Published 2005	Part of large Pacific Rim study comparing practice in different countries. Parents in Melbourne Australia. Babies under 1501 g, mean gestation 29.2 weeks	51 Australian parents response rate unknown	Survey (by structured telephone interview)	74 % felt that physicians had made all resuscitation decisions alone. Majority of babies had done better than expected from the antenatal counseling prognosis. Less than 50 % felt that ante-natal counseling adequate

### Outcome of extreme prematurity

It is difficult to define the current risk of long-term disability in the survivors of the neonatal intensive care unit. Studies often have small numbers of the most premature babies [18], follow the participants for insufficient time for the full extent of the outcome to be clear [19, 20], and use variable definitions of disability [12, 13]. Some report data in relation to birth weight rather than gestation, which allows the inclusion of more mature but lighter infants [21]. In addition, over time, the medical management of babies has changed and the generalizability of outcome studies to an era where management is different is debatable. There is a paucity of very long term studies that reveal how these vulnerable babies fare into adulthood.

The meta-analysis by Saigal and Doyle (2008), who aimed to investigate the long term outcome of extremely preterm babies, found only nine papers which provided sufficient data to analyse. The study babies were all born between 1990 and 1997 and only three studies had followed the babies up beyond two years of age [12]. Each of the studies used a different definition of disability, making comparisons between studies difficult. Definitions varied from ‘cerebral palsy’, to ‘moderate to severe cerebral palsy’ to ‘unable to walk without assistance’. Sensory disability was variably described as ‘unilateral blindness’, or the ‘requirement for hearing aids’, to ‘blind’ and ‘hearing uncorrected with hearing aids’. Not all developmental assessments were performed using standardized psychometric evaluation and thresholds varied from more than ‘two standard deviations from controls or the mean’ to an ‘intelligence quotient of less than 50’. Yet the meta-analysis does demonstrates that, in this era, a significant number of babies had severe handicap with figures ranging from 21 to 35 %. It is interesting to note that the only study which follows the patients up beyond five years of age – in this case to age 11, had the lowest rate of disability despite also having a lower threshold required to include disability by definition [22]. The studies which followed babies for the shortest duration appeared to have the highest rates of disability- an observation that has been noted by a number of authors [9, 20].

A more recent meta-analysis by Moore et al. (2013) [13] included nine papers where babies were followed up to a minimum of eight years of age. Of note is that 80 studies were excluded, primarily because they contained methodological flaws or because the assessments lacked rigor. Highly selective cohorts, data from clinical trials and review articles were excluded. The papers selected included cohort studies, some with term baby controls, a follow up rate of over 65 % and the use of standardized psychometric assessments. Severe disability was uniformly described as an IQ score more than three standard deviations below the mean, non ambulant cerebral palsy and no useful vision and/or hearing despite amplification. These disabilities are likely to leave the person reliant on others for care-giving throughout life. Moderate disability was defined as IQ two to three standard deviations from the mean, ambulant cerebral palsy, little useful vision, or hearing restored by amplification. The pooled data suggested that from 22 to 26 weeks gestation, gestational age made no difference to the rates of severe impairment (approximately 20 %), although the rates of moderate impairment decreased with increasing gestation. The relatively small numbers in the lowest gestation groups limits the reliability of the aggregated statistics leading to wide confidence intervals. Whilst the authors stated follow up to eight years of age in their inclusion criteria, only two studies achieved this. Despite some flaws, this study attempts to provide the highest quality outcome data available to be used clinically when counselling parents. However, there should be some caution in the use of population epidemiology to provide statistical advice to individual parents [23].

The risk of significant disability has lead to well documented ethical concerns about the provision of intensive care to these babies [6, 24, 25]. The ethical concerns surround the issues of sanctity of life, quality of life, the immediate suffering of the extremely preterm baby, and the rights of the parents who will ultimately care for the babies after discharge from hospital. Health care providers may also have concerns about their duty of care to both the baby and the family. Decisions to resuscitate very high-risk babies depend on the country of birth [26, 27] and reflect differences in cultural and religious beliefs. Australia has similar decision-making processes to other developed countries such as the United Kingdom and parts of the United States of America [27]. In these countries, discussions with the parents prior to delivery are considered best practice, with the decision to resuscitate and offer life sustaining interventions weighted towards parental preference at the most extreme age ie 22–23 weeks gestation, but considered to be usually appropriate after 25 completed weeks of gestation. This is based on the increased expectation of intact survival after this gestation and is reflected in the guidelines in use in different states in Australia [28, 29].

### Attitudes of health care providers to extreme prematurity

Clinicians in Australia who care for women at high risk of delivering between 22 and 27 weeks gestation include the primary health team, the midwife and obstetricians. Prior to delivery these women will also come into contact with neonatologists who will care for the baby after delivery, and neonatal nursing staff who will often orientate the parents to the neonatal unit and provide a source of information. Actions by obstetricians prior to a baby’s birth may improve the chance of survival and decrease the rate of complications, improving the future morbidity of these babies. Possible interventions include the administration of antenatal steroids, magnesium sulphate for fetal neuroprotection, and monitoring of the baby with a view to earlier surgical delivery if there are signs of distress [30, 31]. Midwives, neonatologists and neonatal nurses also play a significant role in informing parents about the future for their baby [32–34]. As current guidelines suggest parental participation in decisions around providing or withholding treatment, parental views are important. Message framing by all members of the treating team may have an effect on parental opinion. A study of adult volunteers, who were posed a vignette involving a 23 week gestation baby whose delivery was imminent, showed that those participants who were presented with a positively framed message were significantly more likely to suggest that resuscitation should be provided when compared with those provided with a negatively framed scenario [35]. The clinical facts in both scenarios were identical. This study had a number of limitations in that the participants were not in the emotive situation of being faced with making this decision for their own pregnancies. However, despite these limitations, this study shows that the way information is presented by clinicians (or health care providers) is important and may influence parental decisions.

The attitudes of health care professionals in Australia have been explored in a number of studies [36–40]. Obstetricians from 18 hospitals with a level 3 neonatal unit (able to provide the highest level of neonatal care) were asked to participate in Mulvey et al’s 2001 study about their personal attitudes towards antenatal counselling, resuscitation and the expected survival rates of extremely preterm babies [38]. Obstetricians from 12 units were enrolled in the study with a response rate of 48 % from the clinicians. Responses to hypothetical delivery at different gestations were assessed using a structured questionnaire. From 23 weeks gestation, obstetricians were increasingly likely to discuss resuscitation of the baby with the parents and two thirds said that they would alter the perinatal plan according to parental wishes. It is notable that a third did not include any discussion about the potential death of the baby, or the option to provide only palliative care following delivery. Factors which would influence the counselling given included previous perinatal loss, and concern about the emotional burden of the counselling for the family. Nearly 40 %, however, stated that they had their own personal criteria around gestational age and the presence of anomalies as part of their decision to involve the paediatric staff. Where there was disagreement about resuscitation between clinicians and parents, 49 % felt that the neonatologists should make the decision about resuscitation, 39 % the parents and 8 % felt it should remain in the hands of the obstetricians. In terms of resuscitation, there was a range of responses about the gestational age at which cardiac massage and adrenaline would be considered appropriate for a baby in poor condition at birth. Mulvey et al. asked the obstetricians about their understanding of survival and intact survival at different gestations and compared this to those found by Yu in unpublished outcome data for Victoria in 1997. Respondents significantly underestimated the survival and disability free survival of babies at all gestations with the biggest discrepancies being at 23 weeks gestation. The design of the study restricted participants to pre-set questions and did not allow investigation of the obstetricians rationale for decisions made. The ‘personal reasons’ why individual clinicians might vary their practice could not be ascertained. The response rate might also provide bias as the characteristics of non-responders are unknown.

Gooi et al. explored the attitudes of non-tertiary obstetricians in 2001 [39]. This study also used a structured questionnaire, with repeated postings to ensure a higher response rate. They received a 75 % participation rate of all obstetricians registered in units providing level 2 neonatal services (able to manage babies over 32 week’s gestation) in Australia. Clinicians were asked their opinions about the gestation at which they would consider transfer and active management. They were posed a clinical scenario, given a list of interventions and asked about which intervention they considered appropriate at which gestation. Knowledge about morbidity and mortality was explored. Most would transfer women to a tertiary level hospital prior to extremely preterm delivery, although this would occur from 22 weeks for the West Australian and Victorian clinicians but only after 24 weeks for the rest of the states. The mean age for suggesting administration of steroids was 24 weeks and surgical delivery at 26 weeks gestation. Most respondents underestimated survival, particularly at the lowest gestations with the West Australian and Victorian clinicians being the least pessimistic. 74 % of the obstetricians would involve a paediatrician in antenatal counselling. This study suggested that despite underestimating the outcomes of extremely preterm babies, most clinicians would actively manage and transfer most babies of low gestation. However, where decisions are made by parents, it is likely that the parents being counselled by the clinicians would receive incorrect information and this may affect their decisions. This paper did not have a qualitative component which might have facilitated an understanding of the differences in management seen in different jurisdictions, or the attitudes of the clinicians towards the ethics of resuscitating the extremely preterm baby, which could affect message framing for the parents. Although the obstetricians often asked their paediatric colleagues in level 2 hospitals to consult with the parents, the study made no effort to explore the attitudes of the paediatricians in the same hospitals, and no Australian data was found which evaluated views of non-tertiary hospital paediatricians.

Despite being the initial counsellors of the parents, obstetricians underestimate survival, Australian neonatologists also underestimate survival and disability free survival, although to a lesser degree [36, 40]. A number of studies have investigated the attitudes of neonatologists, with one also including neonatal nurses [40]. De Garis et al. (1987), sent multiple copies of a questionnaire to each neonatal intensive care unit in Australia. They received 51 replies but it is unknown how many neonatologists were in practice at the time, or the units which were represented in the study. Neonatologists were asked about their understanding of mortality and morbidity, treatment at birth for differing gestations, withdrawal of life sustaining interventions, and hospital guidelines. Some open ended questions allowed narrative feedback. They found that the majority of neonatologists would, if called to the delivery of a 24–25 week live baby, invariably initiate resuscitation measures. Others would not do so if the parents were strongly against resuscitation. All, however, would later consider withdrawal of life sustaining interventions where they judged that there was a high probability of severe brain damage, a congenital anomaly which would be problematic, or during the neonatal course where there was irreversible respiratory failure or overwhelming sepsis. Most felt that the withdrawal of life sustaining intervention decisions should be made during a consultative process together with nursing staff and the parents. De Garis commented that if the clinician believes that the baby has little chance of survival, and then withdraws life sustaining interventions, this becomes a self-fulfilling prophecy. This study was done in an era where resuscitation at 22 weeks was not considered at all, and survival was below 33 % for all gestations less than 26 weeks [41]. Although participants were invited to offer comments, there is little reporting in the study of any commentary received. Open questions in this type of study may not produce good qualitative data.

A 1997 study by Oei et al. surveyed all neonatologists, and three registered nurses in each unit, in all neonatal intensive care units in Australia. They asked for opinions about resuscitation at different gestations using 26 graded response questions and three open ended questions. Very high response rates of 93 % and 73 % were received for the doctors and nurses respectively. Over 20 % of neonatologists would occasionally resuscitate 22 week gestation babies and 25 % would often resuscitate a 23 weeker. By 24 weeks, 74 % of neonatologists would almost always resuscitate the baby. Neonatal nurses were much less likely to suggest resuscitation at all gestational ages to 25 weeks, but more likely over 25 weeks. Survival was underestimated by both groups, but more so by the nurses. This reflects the findings of other studies comparing neonatal doctors and nurses [42] and obstetricians and midwives [31]. Doctors accurately reported rates of disability free survival, but not the nurses who underestimated this at all gestations. Eighty-five percent of neonatologists would have a threshold above which they would resuscitate the baby despite parental request not to do so. The mean threshold was 25 weeks, but ranged from 22 to 27 weeks. As these studies are all done between 1997 and 2004 the findings may no longer represent the current opinion of clinicians as medical management has evolved. The paper tabulates comments given by participants but it is not clear if these comments are representative or are all the comments given, and there is no thematic assessment of the comments. This study is interesting in that it suggests that withholding resuscitation seems to be less likely than consideration of withdrawal of life sustaining interventions after resuscitation for those whose prognosis looks worse. A similar study by Munroe et al. published in 2001 suggested that 86 % of neonatologists often/always followed the wishes of parents at 23–25 weeks gestation [37]. This study also used a questionnaire methodology with graded as well as yes/no questions which was sent to 100 neonatologists. The results seem at odds with the paper by Oei et al., where the mean gestation at which parental decisions would be over-ridden was 25 weeks. It must be assumed that the same relatively small group of neonatologists completed both questionnaires as there is only a small pool of neonatologists in Australia, and the response rates in both studies was high. The latter paper suggested that counselling was often based on ‘parents’ perceived wishes’. Again participants underestimate survival. The attitudes of neonatologists in Australia are reaffirmed in the study of practice in Pacific Rim countries by Martinez et al. (2005) [43]. This survey study done in 1999 consisted of questions rated on a Likert scale and received a 68 % response from Australian neonatologists. The purpose of this study was to compare the attitudes of clinicians in different Pacific Rim countries, but there is sufficient data to assess the Australian response. This is the only study which differentiates between different components of resuscitation and showed that the mean age for intubation alone was 22 weeks, use of cardiopulmonary resuscitation from 24 weeks and adrenaline over 24 weeks. Concerns about poor quality of life, parental wishes, congenital anomaly and probable death were major factors in determining resuscitation decisions for individual babies. This study includes a more extensive range of factors which the clinician might take into account. Unfortunately, however, a questionnaire is only able to assess the set factors, which are included by the researcher, and the lack of any qualitative component, renders the participant unable to contribute their individual perspectives or beliefs.

### Attitudes of parents to extreme prematurity

Internationally, parents have been asked about the role they feel they should have in the decision to resuscitate and care for their periviable baby. These studies explored the role of the parents both in the initial resuscitation of the baby as well as the withdrawal of life sustaining interventions when care is considered futile [16, 44, 45]. These international studies suggest that parents themselves do want to be involved in decisions regarding the care of their infants but often do not want to be seen as the primary decision maker. This seems at odds with the guidelines used by clinicians [28, 29, 46] in Australia and the UK where parental choice is said to inform resuscitation at 22–24 weeks gestation.

Studies which look at the overall experience of parents, are usually done among families who have experienced delivery of either very low birth weight babies (below 1000 g) or early gestation. These show that the birth of these very vulnerable babies causes considerable trauma to the family in the acute neonatal period [47–50], followed ultimately by ‘stoic survival’ and for many parents adaptation in the longer term regardless of the wellbeing of the surviving child [51, 52]. However, some studies show a much more difficult long-term experience for parents where children have severe disability [44, 53]. In these qualitative studies, a number of parents reflect that the quality of life for the child is so poor that it might have been better had they not been offered care at all. This is a theme reflected by a number of authors in both the medical literature [54], and media [55] who themselves have given birth to extreme preterm babies.

The Australian literature on parental experience in extreme prematurity is scant. Partridge et al. reported the experiences of 51 Melbourne based parents in a study comparing parental attitudes in the Pacific Rim [26]. This study identified parents who had delivered a baby under 1501 g birth weight in 6 countries. The Australian component enrolled only parents who had received care in Melbourne. This confirmed that 74 % of the Australian parents who had received antenatal counselling felt that the health professionals alone had made all the decisions about the care of the child, and that, as parents, they would not wish to have had to make a decision to withhold care. 74 % felt satisfied with the physician counselling that they had, yet whilst disability was adequately discussed, death was not. Issues of pain, bonding and attachment were also topics that they felt were not discussed adequately. It is interesting to note that most parents felt that their child had progressed much better than they had expected based on the antenatal counselling that they had received. This may be explained by the under-estimation of outcomes which was described in the study of clinician understanding of outcomes by Martinez [43]. The majority of this group of babies was of a gestation older than would currently be considered periviable, so it is likely that the ethics of periviable care would not be relevant. The mean gestation of this cohort was over 29 weeks and 29 % were described as having sequelae although the functional outcomes for the babies are not known as this was merely assessed by the presence of neonatal complications. Although the participants were interviewed by telephone, the researchers used a structured questionnaire with fixed questions and all answers were given on a Likert scale. Open-ended questions were only asked about the nursery experience of the participants. This study has the potential for recruitment bias as parents were invited to participate and the total number of eligible parents is unknown. The usefulness of this study in a narrative on periviable babies in North Queensland is questionable. It is, however one of the few studies available on this topic.

### The North Queensland perspective

Periviable babies in The Townsville Hospital come from families throughout the North Queensland region, and also occasionally from further afield when, for example, holiday makers unexpectedly deliver very early. The neonatal unit is the only tertiary neonatal unit in North Queensland and services both the public and private sectors. 74.5 % of 157 babies who were admitted to The Townsville Hospital neonatal unit under 26 weeks gestation from January 2004 to December 2013 had an address outside Townsville city. Aboriginal and Torres Strait Islander mothers account for 30 % of admissions [56]. Despite the large numbers of babies from more regional, rural and remote places, and high proportion of Indigenous babies, which are all risk factors for a poor outcome [57, 58], survival rates from 2008 to 2013, compare well with other major centers. Survival was over 50 % at 23 weeks gestation increasing to 90 % at 26 weeks gestational age.

The health statistics branch of Queensland Health report that in Queensland Indigenous mothers are 4.2 times more likely to be under 20, 3.8 times more likely to attend less than five antenatal visits, 12 times more likely to live remotely or very remotely and 3.6 times more likely to be smoking after 20 weeks gestation than non-Indigenous mothers [58]. In addition they are 1.7 times more likely to deliver before 37 weeks gestation. The risk of neonatal death for Indigenous babies is 2.7 times that for non–Indigenous babies. Prematurity was found to be the strongest predictor of neonatal death in all groups. Queensland Health Statistics confirm the high rate of low birth weight seen in Indigenous groups as found by Kandasamy et al. [59] who have investigated the rate of low birth weight and/ or small for gestational age (SGA) in term babies in Townsville. They found that 20.2 ± 5.7 % of Aboriginal or Torres Strait Islander babies had low birth weight as opposed to 10.2 +/− 1.9 % for non-Indigenous babies.

Very little is known about the experience of women who deliver a preterm baby in regional, rural or remote parts of Australia, and particularly about Aboriginal and Torres Strait Islander women. Australia is a large and geographically diverse country. Outcomes for babies from outside urban areas are worse than those from the urban areas [57]. Coory, in his 2003 paper [60] based on routine perinatal data collection in Queensland, suggested that the excess neonatal mortality found in rural and remote Australia is entirely accounted for by a high level of mortality in Aboriginal and Torres Strait Islander populations which is found regardless of place of residence. A higher proportion of the Aboriginal and Torres Strait Islander population lived in rural and remote areas leading to the difference between urban and non-urban sites. He found that non-Indigenous babies from rural and remote areas had no excess perinatal mortality when compared to their urban counterparts. Steenkamp et al. [61] studied births in the Northern Territory of Australia by ethnic classification of the mother and also the remoteness of maternal address. They found that Indigenous women in remote areas had more antenatal risk factors then non-Indigenous women, and their babies had a worse outcome. For Indigenous women, increasing remoteness was associated with worsening outcomes. Their study, unlike the study by Abdel-Latif et al., did not show any increase in mortality in non-Indigenous women related to place of residence, which supports the findings of Coory. The majority of the babies in these two epidemiological studies were born at term, and comparison of the findings for ethnicity and usual place of residence for premature babies was sought.

Abdel-Latif et al. (2006) [57] studied major morbidity and mortality in premature babies born in NSW and ACT from 1992–2002. Babies born in the non-urban centers had the highest mortality, but even when born in the tertiary centre, the babies born to women with a non-urban address did less well. They found that women from rural areas were more likely to be Aboriginal, teenaged or have a previous preterm birth. Prolonged rupture of membranes and spontaneous labour heralded the prematurity. Urban women, however, were more likely to be older, had assisted conception and have multiple births. An antenatal diagnosis of intra uterine growth retardation and delivery by Caesarean section were also more common in urban women. Despite a higher mortality, the rate of serious morbidity during the neonatal period was the same for both groups of babies. This analysis of the characteristics of the rural women showed an increase in relative prenatal disadvantage in comparison to the urban women. The paper further references the poorer health outcomes of people living in rural and remote areas, including a higher rate of stillbirth.

Only one paper was found which investigated families from a rural area who had the experience of a preterm baby in a neonatal intensive care unit [52]. The investigators recruited seven parents from five families in rural NSW who had delivered babies between 26 and 34 weeks gestation, with a median gestation of 32 weeks. Only one child was described as having a significant disability. The families in this phenomenological study described the initial traumatic phase of hospitalization as one of shock and confusion leading to acceptance of their situation. The transfer and stay in a metropolitan center far from home resulted in leaving other children behind for a period of time. There were financial burdens and concerns about leaving properties untended. After adapting to the metropolitan environment, transfer back to the local hospital was a time of anxiety with concerns that the local hospital may not be able to meet the level of care their child required. At interview some time later, the families felt that receiving medical care in the local area gave them improved access to local services and allowed clinicians to get to know the children well on a more personal level. The themes identified in the paper were those of ‘coping through optimism’ in the early days of hospitalization, ‘stoic survival’ where families were unable to discuss their true emotional turmoil with anyone else, followed by ‘striving for normal’ where developmental achievements were celebrated and delays were devastating. The limitations to this study may reduce its transferability to North Queensland in that the distance from the metropolitan areas was considerably less than that of many of the Townsville neonatal unit patients. Aboriginal patients were specifically excluded. Lastly, the babies were of a gestation where full medical care was not an ethical issue for all but one. The study is, however, of interest as it suggests that families from rural areas may have challenges related to their place of residence which are not experienced by urban families.

Following discharge from hospital, the high risk baby will need follow up and monitoring for developmental delay which may be problematic in areas where there are workforce difficulties in recruitment and retention in allied health [62]. Developmental assessment tools which are based on parental self report have been found to give an inaccurate assessment of the development in some babies especially for remote Aboriginal babies where the testing is neither culturally appropriate nor validated for these populations [63]. Children with identified disabilities are provided with services for early intervention in order to help reduce the functional limitation the disability poses. Rural families have less availability of services and less choice in services they can access [64]. Transport is frequently a problem [65, 66]. There has been an attempt to use videoconferencing for routine specialist appointments such as genetic and orthopaedic reviews [67, 68], which have generally been satisfactory. In addition to the chronic burden of prematurity, acute illness is also more common in babies who have been extremely premature and this will often necessitate transfer to urban or tertiary level services [69]. This further adds to the burden for the rural family caring for a baby who was periviable. The additional burden of caring for a high risk baby after discharge may be great for many families.

### Conclusion/Discussion

This review has explored the literature around the outcomes of extreme prematurity, and the attitudes of clinicians and families to the extremely preterm baby in Australia. The literature reviewed suggests that Australian clinicians, particularly obstetricians and neonatologists have been the decision makers who determine which babies will be resuscitated and which will not, although the importance of parental opinion was stated. It is clear that the clinicians underestimated the outlook for extremely preterm babies, yet what informs the clinicians’ decision-making is not clear. The studies done are all over 10 years old and given the changing nature of neonatal intensive care and the improvement in outcomes, may not reflect current opinion.

Parental decision-making is suggested as being of primary importance in all the current resuscitation guidelines at the extremes of periviability. This assumes that parents are in the best position to make a decision for their baby in the role of surrogate decision maker. It assumes that parents are adequately informed and competent to make these decisions. If, however, the counselling clinician is ill informed and has personal bias in their message framing, parents may not be able to accurately assess their options. Research is required to ascertain whether parents in Australia want this burden of choice or not, and how this knowledge might improve clinicians use of the decision making process in preterm babies. Parents who have experienced a baby born on the verge of viability may be well placed to inform the discussion on whether resuscitation has been appropriate for their families. The realities of their lived experiences, whether they are in a metropolitan area or the more remote areas of Australia have not been heard.

In order for clinicians to understand the consequences of resuscitation for families, families who have lived through periviable births need to be able to voice their experiences. This must inform clinician knowledge and hence counselling of future parents in a similar situation. In addition, the reality and process of clinician-lead decision making and theoretical proposed parental choice needs to be further explored.
